# 590. A New Thin-Film Transistor Image Sensor for Estimation of Bacterial Colony Species on Agar Plates

**DOI:** 10.1093/ofid/ofad500.659

**Published:** 2023-11-27

**Authors:** Mitsutaka Nakada, Tsubasa Inagaki, Reiichi Ariizumi, Shogo Maeta, Hiroaki Ozaki, Akihiko Fujisawa, Tomoya Tezen, Takanori Tsunashima, Kaoru Ito, Daichi Abe, Kazunori Yamaguchi, Masakazu Nakajima, Makoto Taketani

**Affiliations:** CarbGeM Inc., Kobe, Hyogo, Japan; CarbGeM Inc., Kobe, Hyogo, Japan; CarbGeM Inc., Kobe, Hyogo, Japan; CarbGeM Inc., Kobe, Hyogo, Japan; CarbGeM Inc., Kobe, Hyogo, Japan; Japan Display Inc., Ebina-shi, Kanagawa, Japan; Japan Display Inc., Ebina-shi, Kanagawa, Japan; Japan Display Inc., Ebina-shi, Kanagawa, Japan; Japan Display Inc., Ebina-shi, Kanagawa, Japan; Japan Display Inc., Ebina-shi, Kanagawa, Japan; Japan Display Inc., Ebina-shi, Kanagawa, Japan; CarbGeM Inc., Kobe, Hyogo, Japan; CarbGeM Inc., Kobe, Hyogo, Japan

## Abstract

**Background:**

Early detection and identification of pathogenic bacteria is an important public health issue. Conventional methods of culturing specimens on agar plates usually take overnight to obtain definitive results. Here, we developed and verified the performance of a new thin-film transistor (TFT) image sensor for estimating bacterial colony species on agar plates. This system uses transmitted light, unlike conventional image analysis using reflected light.

**Methods:**

To demonstrate the efficacy of this colony species estimation system, a lens-free imaging modality was built using the TFT image sensor consisting of a control printed circuit board, an image sensor array (542 x 872 pixels, pixel size = 80 μm) and flat surface illuminating module. The field of view (FOV) of 44 x 70 mm can cover 55% of the area of the 90 mm petri dish. Each pixel measures the intensity of light passing through the agar medium.

Verification of the sensor was conducted using *Enterobacter cloacae* (GTC21793), *Escherichia coli* (ATCC BAA-2452), and *Klebsiella pneumoniae* (ATCC BAA-1705) spread on chromogenic agar medium. Images of bacterial colonies cultured on agar plates were automatically collected at 5 min intervals. Triplicate experiments were conducted on each of the bacteria. One was used as the test dataset and the other two as the correct labels dataset. Colony images were produced by removing the background (medium region) and extracting only the bacterial regions. The color histograms of the test data and the correct data were compared, and the labels of the correct data with the highest similarity (correlation coefficient) were used as inferred labels for the test data (Fig 1).

**Figure 1.**
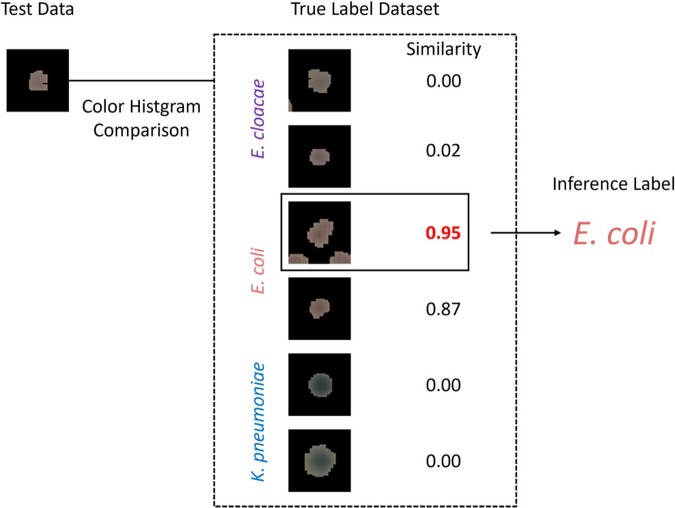
Inference scheme for test data by color histograms comparison.

**Results:**

Comparisons using RGB channels showed 100% accuracy (Fig 2). In comparisons using HSV-transformed H (Hue) and S (Saturation) channels, the accuracy was also 100% (Fig 3).

**Figure 2.**
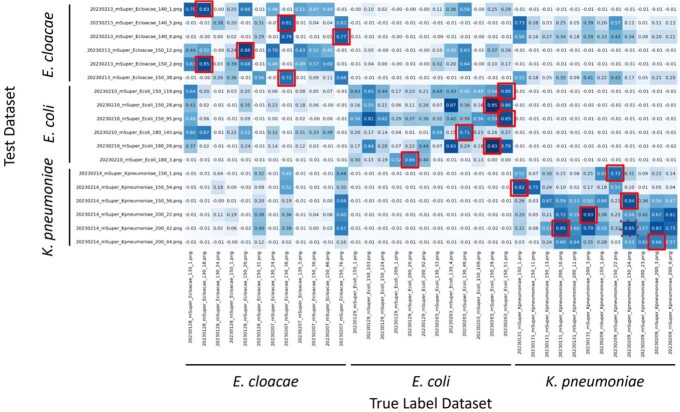
Bacterial species estimation by color (RGB) histograms comparison

The vertical axis shows test data and the horizontal axis shows correct data. The numbers indicate the correlation coefficient between the test data and the correct label data. Red boxes indicate those with the highest similarity to the correct dataset.

**Figure 3.**
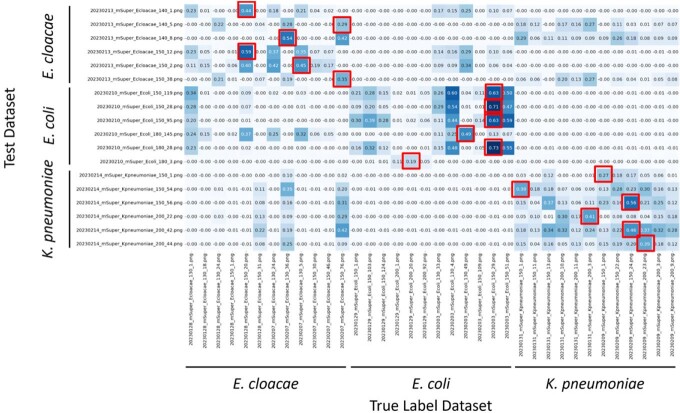
Bacterial species estimation by color (Hue, Saturation) histograms comparison

The vertical axis shows test data and the horizontal axis shows correct data. The numbers indicate the correlation coefficient between the test data and the correct label data. Red boxes indicate those with the highest similarity to the correct dataset.

**Conclusions:** The new TFT image sensor was able to estimate bacterial colony species on agar plates. Furthermore, it was shown that a simple rule-based algorithm of comparing color histograms can be used to estimate the bacterial species of colonies on agar media without the use of complex systems such as deep learning. We will also work to conduct experiments on other media and bacterial species.

**Disclosures:**

**Masakazu Nakajima, B. Engineering**, Soiken Holdings: Board Member|Welby Inc.: Board Member|Welby Inc.: Ownership Interest|Welby Inc.: Stocks/Bonds

